# Supplementary material for: Altruistic feeding and cell-cell signaling during bacterial differentiation actively enhance phenotypic heterogeneity
Source: bioRxiv. 2024 Mar 27:2024.03.27.587046. Preprint. [Version 1] doi: 10.1101/2024.03.27.587046 (PMC11188070; doi:10.1101/2024.03.27.587046)
Supplement: Supplement 1 [file NIHPP2024.03.27.587046v1-supplement-1.pdf]

bioRxiv preprint doi: https://doi.org/10.1101/2024.03.27.587046; this version posted March 27, 2024. The copyright holder for this preprint (which was not certified by peer review) is the author/funder. This article is a US Government work. It is not subject to copyright under 17 USC 105 and is also made available for use under a CC0 license.

SUPPLEMENTAL INFORMATION

(Figures S1-S3)

bioRxiv preprint doi: https://doi.org/10.1101/2024.03.27.587046; this version posted March 27, 2024. The copyright holder for this preprint (which was not certified by peer review) is the author/funder. This article is a US Government work. It is not subject to copyright under 17 USC 105 and is also made available for use under a CC0 license.

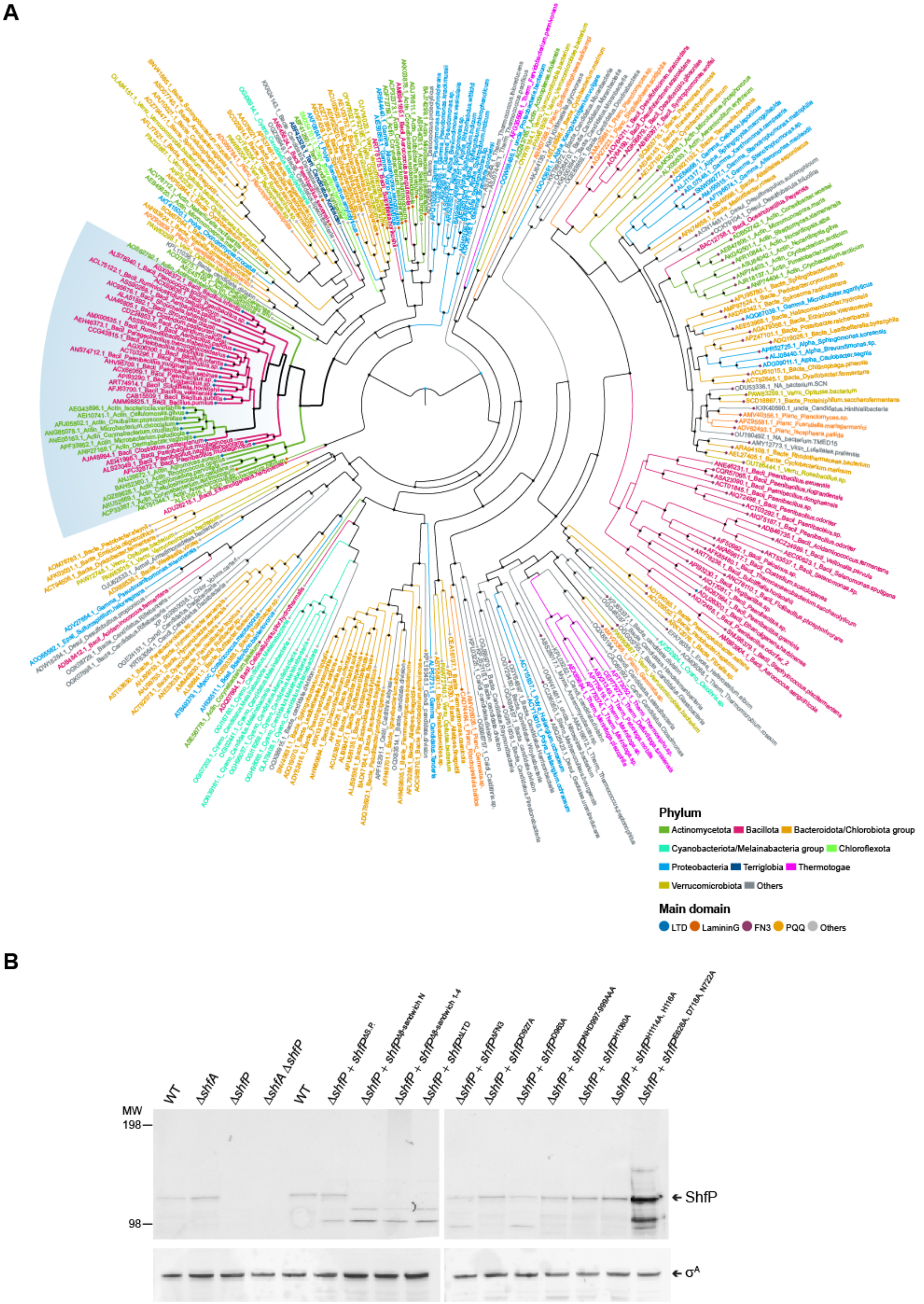

Figure S1. Phylogenetic analysis of ShfP and calcineurin-like proteins. (A) A phylogenetic tree of a representative set of extracellular calcineurin-like domains from the homologs of the ShfP family is shown. The name of each member is shown with its accession number, phylum, and organism name. (B) Western blot against ShfP (top panel) for the following strains: WT (lane 1); Δ*shfA* mutant (lane 2); Δ*shfP* mutant (lane 3);

bioRxiv preprint doi: https://doi.org/10.1101/2024.03.27.587046; this version posted March 27, 2024. The copyright holder for this preprint (which was not certified by peer review) is the author/funder. This article is a US Government work. It is not subject to copyright under 17 USC 105 and is also made available for use under a CC0 license.

Δ*shfA* Δ*shfP* mutant (lane 4); Δ*shfA ΔshfP amyE::shfP* (lane 5); Δ*shfA ΔshfP amyE::shfP* (*Δ2-28)* (lane 6); Δ*shfA ΔshfP amyE::shfP (Δ27-139)* (lane 7)*;* Δ*shfA ΔshfP amyE::shfP(Δ293-732)* (lane 8); Δ*shfA ΔshfP amyE::shfP(Δ140-280)* (lane 9); Δ*shfA ΔshfP amyE::shfP(Δ1154-1289)* (lane 10); Δ*shfA ΔshfP amyE::shfP(D927A)* (lane 11); Δ*shfA ΔshfP amyE::shfP(D963A)* (lane 12); Δ*shfA ΔshfP amyE::shfP(NHD997-999AAA)* (lane 13); Δ*shfA ΔshfP amyE::shfP(H1080A)* (lane 14); Δ*shfA ΔshfP amyE::shfP(H114A, H116A)* (lane 15); Δ*shfA ΔshfP amyE::shfP(E628A, D718A, N722A)* (lane 16). Anti-sigA used as a loading control (bottom panel). Strains: PY79; CW202; TD517; TD507; CC2; CC19; CC131; CC20; CC138; CC14; CC15; CC16; CC17; CC18; CC166.

bioRxiv preprint doi: https://doi.org/10.1101/2024.03.27.587046; this version posted March 27, 2024. The copyright holder for this preprint (which was not certified by peer review) is the author/funder. This article is a US Government work. It is not subject to copyright under 17 USC 105 and is also made available for use under a CC0 license.

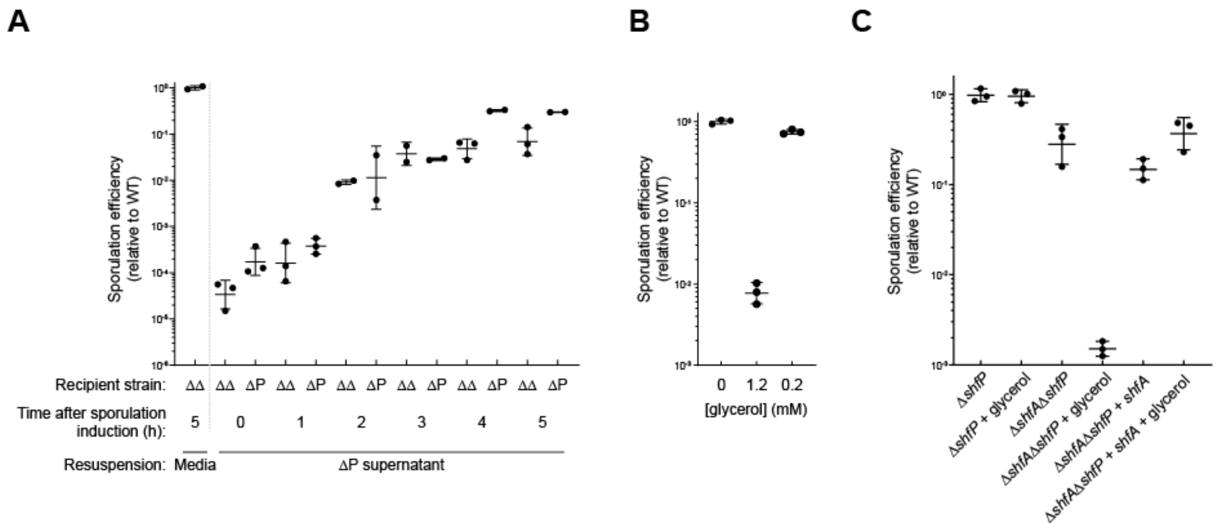

Figure S2. Extracellular glycerol inhibits sporulation. (A) Cell-free supernatant derived from the *ΔshfP* mutant strain show no sporulation suppression activity. Sporulation efficiency of the *ΔshfA ΔshfP* (TD507) and *ΔshfP* (TD517) mutant recipient strains cultured for the indicated times in synchronous sporulation media prior to the addition of cell-free supernatants derived from the *ΔshfP* mutant strain. Sporulation efficiencies are reported as normalized values to that of the recipient strain cultured in the absence of supernatant. Mean and standard deviation of two or more independent cultures for each condition are shown. (B) The addition of glycerol to sporulation media causes a sporulation defect. Average sporulation efficiency of the *ΔshfA ΔshfP* (TD507) mutant strain cultured in synchronous sporulation media with the indicated amount of glycerol added. Sporulation efficiencies are reported as normalized values to that of the recipient strain cultured in the absence of glycerol. Mean and standard deviation of three independent cultures for each condition are shown. (C) Sporulation efficiencies of the *ΔshfP* (TD517), *ΔshfA ΔshfP* (TD507), and *ΔshfA ΔshfP thrC::shfA* (CC221) strains cultured in synchronous sporulation media in the presence or absence of 1.3 mM glycerol. Sporulation efficiencies are reported as normalized values to that of the *ΔshfP* (TD517) recipient strain cultured in the absence of glycerol. Bars represent mean; errors: S.D.

bioRxiv preprint doi: https://doi.org/10.1101/2024.03.27.587046; this version posted March 27, 2024. The copyright holder for this preprint (which was not certified by peer review) is the author/funder. This article is a US Government work. It is not subject to copyright under 17 USC 105 and is also made available for use under a CC0 license.

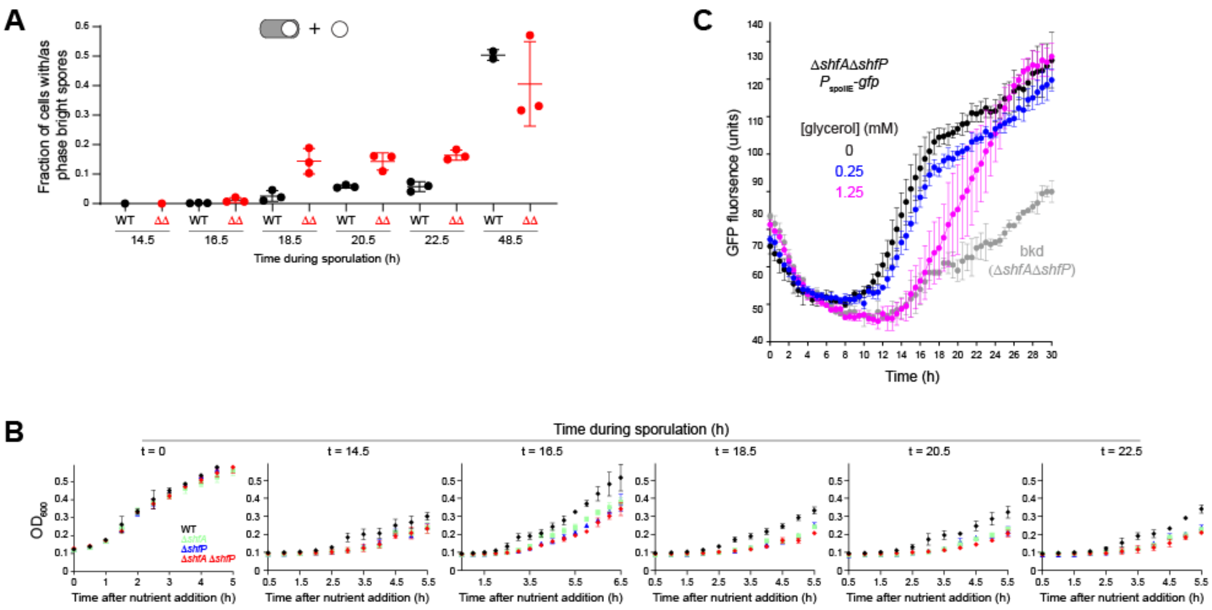

Figure S3. Glycerol secretion delays formation of DIC-bright cells (A) Fraction of (black) WT (MF277) or (red) ∆*shfA* ∆*shfP* (CC219) cells forming DIC-bright forespore or mature spore structures (as shown in the cartoon depictions above) at the indicated time points. Bars represent mean; errors: S.D.; data points represent a measure from an independent experiment. (B) WT cells respond faster to nutrient replenishment than ∆*shfA* ∆*shfP* mutant cells when grown under asynchronous sporulation conditions. WT (MF277), ∆*shfA* (CC218), ∆*shfP* (CC220) and ∆*shfA* ∆*shfP* (CC219) mutant strains were grown in asynchronous sporulation media. At the indicated time points, 10% of each culture was back diluted in fresh nutrient rich LB media and the OD_{600} was measured over time. Mean and standard deviation of three independent cultures are shown. The initial doubling time for each strain at the indicated time points of subculturing were used for Fig 5B. (C) The addition of glycerol to sporulation media causes a delay in entry into sporulation. The *∆shfA ∆shfP* P_{spoIIE}*-gfp* (CC219) reporter strain and the non-gfp fusion strain *∆shfA ∆shfP* (TD507) were grown in synchronous sporulation media with the indicated amount of glycerol added. GFP fluorescence was recorded over time (hours). Mean and standard deviation of four or more independent cultures for each strain and condition are shown.
